# An unusual presentation of brucellosis, involving multiple organ systems, with low agglutinating titers: a case report

**DOI:** 10.1186/1752-1947-1-53

**Published:** 2007-07-21

**Authors:** Farzin Khorvash, Ammar H Keshteli, Mohaddeseh Behjati, Mansoor Salehi, Alireza Emami Naeini

**Affiliations:** 1 Department of Infectious and Tropical Diseases, Alzahra University Hospital, School of Medicine, Isfahan University of Medical Sciences, Isfahan, Iran

## Abstract

**Background:**

Brucellosis is a multi-system disease that may present with a broad spectrum of clinical manifestations. While hepatic involvement in brucellosis is not rare, it may rarely involve the kidney or display with cardiac manifestations. Central nervous system involvement in brucellosis sometimes can cause demyelinating syndromes. Here we present a case of brucella hepatitis, myocarditis, acute disseminated encephalomyelitis, and renal failure.

**Case presentation:**

A 26-year-old man presented with fever, ataxia, and dysarthria. He was a shepherd and gave a history of low grade fever, chilly sensation, cold sweating, loss of appetite, arthralgia and 10 Kg weight loss during the previous 3 months. He had a body temperature of 39°C at the time of admission. On laboratory tests he had elevated level of liver enzymes, blood urea nitrogen, Creatinine, Creatine phosphokinase (MB), and moderate proteinuria. He also had abnormal echocardiography and brain MRI. Enzyme-linked immunosorbent assay for IgG and IgM was negative. Standard tube agglutination test (STAT) and 2-mercaptoethanol (2-ME) titers were 1:80 and 1:40 respectively. Finally he was diagnosed with brucellosis by positive blood culture and the polymerase chain reaction for Brucella mellitensis.

**Conclusion:**

In endemic areas clinicians should consider brucellosis in any unusual presentation involving multiple organ systems, even if serology is inconclusive. In endemic areas low STAT and 2-ME titers should be considered as an indication of brucellosis and in these cases additional testing is recommended to rule out brucellosis.

## Background

Brucellosis is still an important public health problem and endemic in many countries, especially in Mediterranean areas, parts of south and Central America, and east and western Africa [1]. It is the most common zoonosis in the world; accounting for the annual occurrence of more than 500,000 cases [2]. Brucellosis is a systemic disease and may involve any organ system.

Here, we present a patient with brucella hepatitis, myocarditis, acute disseminated encephalomyelitis, and renal failure with low agglutinating titers.

## Case presentation

A 26-year-old man referred to our center (Alzahra University Hospital, Isfahan, Iran) with complaints of fever, ataxia and dysarthria. His problem began 3 months prior to the recent hospitalization with low grade fever, chilly sensation, cold sweating, loss of appetite and 10 Kg weight loss. He was also complaining from arthralgia in different joints and could not walk without help. His dysarthria and vomiting started 10 and 2 days before the admission, respectively. He had undergone left nephrectomy for congenital urethropelvic junction obstruction and severe hydronephrosis 2 years ago. He was symptom free up to the recent referral.

Physical examination revealed an ill looking young man with body temperature of 39°C, pulse rate 45/Min, and blood pressure of 110/60 mm Hg. He was dysarthteric and truncal ataxia was also observed. On auscultation he had muffled heart sounds without any murmur. The abdomen was soft with tender hepatomegaly 3 cm below the costal margin.

Results of laboratory tests made on admission were as follow: White blood cell count: 17300/mm^-3 ^with 91%Neutrophils, Patelet count: 89000/mm^-3^, Hemoglobin: 14.1 g/dl, C-reactive protein: 15 mg/dl, Erythrocyte sedimentation rate: 41 mm/h, Blood urea nitrogen(BUN): 40 mg/dL, Creatine(Cr): 3.9 mg/dL, SGOT: 338 mg/dL, SGPT: 151 mg/dL, Alkaline phosphatase: 593 mg/dL, Creatine phosphokinase(CPK): 468 mg/dL, CPK(MB): 38 mg/dL,. Urinalysis revealed moderate hematuria and 24-hour urinalysis (U.A) revealed proteinuria (1143 mg/dL). ANA, ANCA, anti-dsDNA, RF, Anti Cardiolopin Ab, Lupus Anticoagulant were all negative. A PPD skin test was negative.

**Figure 1:**
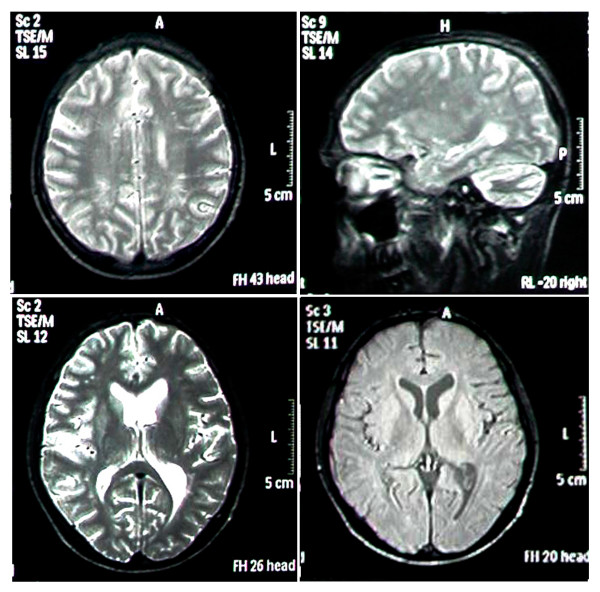
Brain MRI showing white matter changes (hyperintensities) in different parts such as periventricular area, thalamus, and centrum semiovalis.

Chest X ray was normal. Electrocardiogram showed sinus bradycardia and high T waves in the precordial leads. Echocardiographic examination revealed septal hypokinesia and ejection fraction of 30%. No vegetation was reported. Brain MRI showed white matter changes in corpus callosum, periventricular area and centrum semiovalis (Fig. 1). Liver and kidney biopsies as well as lumbar puncture were not performed due to our patient's refusal.

Since he was a shepherd from an endemic area for brucellosis and gave a history about the consumption of non-pasteurized dairy products, standard tube agglutination test (STAT), 2-mercaptoethanol (2-ME), and Coombs test were performed (Antigen from the Pasteur Institute of Iran, Tehran). STAT and 2-ME titers were 1:80 and 1:40 respectively. Titer of Coombs test was 1:80. Enzyme-linked immunosorbent assay (IBL, Germany) for IgG and IgM was negative. Blood culture was performed once and it was positive for brucellosis. The *Brucella omp2a *gene was used as target DNA for PCR amplification. Polymerase chain reaction-restriction fragment length polymorphism (PCR-RFLP) [5] demonstrated the presence of Brucella mellitensis bv 1. Doxycycline (2 × 100 mg/day p.o.) and Rifampicin (1 × 600 mg/day p.o.) were started. The patient received this treatment for six weeks. One week after the initiation of the treatment our patient's symptoms subsided and he became afebrile. After 2 weeks he had normal liver function tests, BUN, and Cr levels and transthoracic echocardiography was normal too. The patient was followed-up for 10 months. In 6^th ^months he experienced a relapse of brucellosis with signs and symptoms similar to the previous episode of the infection. The standard treatment regimen was started again. He is now receiving this treatment and is asymptomatic but still dysarthric. Our plan is to continue the medication for at least 6 months.

## Discussion

Hepatic involvement in brucellosis is not rare and hepatomegaly may be documented in 15–20% of cases [3]. However, liver function tests are usually mildly elevated [1,3]. The spectrum of pathological findings in brucella hepatitis is varied. Granulomatous hepatitis, diffuse hepatitis without granuloma and focal necrosis have been reported [4]. Our patient was diagnosed as having brucella hepatitis because of extreme elevation of the hepatic enzymes that subsequently returned to the normal value with treatment.

The central nervous system is involved in 5–7% of cases and such complications often have an ominous prognosis. Meningitis, encephalitis, meningoencephalitis, meningovascular disease, brain abscesses, and demyelinating syndromes have all been reported [5]. We suggest our patient may be a case of neurobrucellosis too. Unfortunately lumbar puncture was not performed because he did not give permission for that. Brain MRI findings were consistent with acute disseminated encephalomyelitis (ADEM), and his truncal ataxia resolved after the treatment. The resolution for dysarthria was partial. The duration of therapy for neurobrucellosis is generally prolonged, varying from 1 to 19 months. Thus we believe our patient has been mismanaged at first and this may be the reason for the relapse of the infection.

Brucellosis may, however, rarely involve the kidney causing acute interstitial nephritis, pyelonephritis, and IgA nephropathy which may cause proteinuria, hematuria, and pyuria. It may also cause caseating granulomas and calcifications [6]. Patients with brucella glomerulonephritis almost always have urinary sediment abnormalities, proteinuria, and/or azotemia, but exact diagnosis is generally established with resolution of the clinical findings after antibiotic treatment for brucellosis [7]. In this case, microscopic hematuria, proteinuria, and increased BUN and Cr levels were detected during the initial laboratory analysis. Renal biopsy was not performed which might have helped in diagnosing the type of renal involvement. In our patient, renal function tests reached base line and U.A abnormalities resolved after the treatment.

Our patient is a case of brucella myocarditis too. Cardiac complications from brucellosis are unusual, occurring in 0–2% of patients and usually manifest as endocarditis [8]. Myocarditis is a rare complication of adult brucellosis and in most instances it is in electrocardiographic and/or echocardiographic diagnosis [9]. Our patient had abnormal echocardiographic findings and elevated levels of CPK (MB), which is due to myocyte necrosis in myocarditis.

Our patient reported a disease history of about 3 months. Whilst it has been shown that patients with subacute brucellosis usually present with relatively high agglutinating antibody titers [10], the agglutinating antibody titers in our patient were low.

The diagnostic criteria for brucellosis in endemic regions are a titer ≥ 1:320 in STAT and/or a titer ≥ 1:160 in 2-ME [11]. The absolute diagnosis of brucellosis requires isolation of the bacterium from blood or tissue samples [5]. While our patient is a definite case of brucellosis by blood culture and PCR, according to the above criteria he doesn't have brucellosis for low titers of serologic tests.

## Conclusion

In endemic areas clinicians should consider brucellosis in any unusual presentation involving multiple organ systems, even if serology is inconclusive. In endemic areas low STAT and 2-ME titers should be considered as an indication of brucellosis and in these cases additional testing is recommended to rule out brucellosis.

## Authors' contributions

FK wrote the manuscript and was involved with the patient's management. AHK participated in the clinical care of the patient and the writing of the case report. MB wrote the manuscript and was involved with the patient's management. MS performed blood culture and PCR of the patient. AEN wrote the manuscript and was involved with the patient’s management. All authors read and approved the final manuscript.

## Competing interests' declaration

The author(s) declare that they have no competing interests.

## References

[B1] Young EJ Mandell GL, Bennett JE, Dolin R Brucella species Principles and Practice of Infectious Diseases 2000 5 New York: Churchill Livingstone 2386 93

[B2] Pappas G Papadimitriou P Akritidis N The new global map of human brucellosis Lancet Infect Dis 2006 6 2 91 9 10.1016/S1473-3099(06)70382-6 16439329

[B3] Sunmez S Cagatay A Karadeniz A Ozsut H Eraksoy H Calangu S A case of acute hepatitis due to brucellosis South Med J 2006 99 632 3 10.1097/01.smj.0000220845.13081.a0 16800426

[B4] Aygen B Sumerkan B Doganay M Sehmen E Prostatitis and hepatitis due to Brucella melitensis: a case report J Infect 1998 36 111 2 10.1016/S0163-4453(98)93486-7 9515679

[B5] Pappas G Akritidis N Bosilkovski M Tsianos E Brucellosis N Engl J Med 2005 352 2325 36 10.1056/NEJMra050570 15930423

[B6] Odeh M Oliven A Acute brucellosis associated with massive proteinuria Nephron 1996 72 688 9 8730444 10.1159/000188962

[B7] Ustun I Ozcakar L Arda N Duranay M Bayrak E Duman K Atabay M Cakal BE Altundag K Guler S Brucella glomerulonephritis: case report and review of the literature South Med J 2005 98 1216 7 10.1097/01.SMJ.0000163307.87372.38 16440924

[B8] Garcia de Lucas MD Castillo Dominguez JC Martinez Gonzalez MS Brucella myopericarditis Rev Esp Cardiol 2004 57 709 10.1157/13064200 15274859

[B9] Jubber AS Gunawardana DR Lulu AR Acute pulmonary edema in Brucella myocarditis and interstitial pneumonitis Chest 1990 97 1008 9 10.1378/chest.97.4.1008 2323233

[B10] Irmak H Buzgan T Evirgen O Akdeniz H Demiroz AP Abdoel TH Smits HL Use of the Brucella IgM and IgG flow assays in the serodiagnosis of human brucellosis in an area endemic for brucellosis Am J Trop Med Hyg 2004 70 6 688 94 15211015

[B11] Roushan MR Amin MJ Abdoel TH Smits HL Application of a user-friendly Brucella-specific IgM and IgG antibody assay for the rapid confirmation of Rose Bengal-positive patients in a hospital in Iran Trans R Soc Trop Med Hyg 2005 99 744 50 10.1016/j.trstmh.2005.02.009 16095642

